# High-resolution magnetic resonance vessel wall imaging in extracranial cervical artery dissection

**DOI:** 10.3389/fneur.2025.1536581

**Published:** 2025-02-25

**Authors:** Sai Shao, Guangbin Wang

**Affiliations:** Department of Radiology, Shandong Provincial Hospital Affiliated to Shandong First Medical University, Jinan, China

**Keywords:** cervical artery dissection, vessel wall imaging, black blood, ischemic stroke, intramural hematoma

## Abstract

Extracranial cervical artery dissection (eCAD) is the second leading cause of stroke in young and middle-aged adults. Clinical management strategies for eCAD are continuously being explored and optimized, as revealed by the recently published CADISS and TREAT-CAD studies. The type of drug, dosage, and timing of administration can affect the regression of carotid artery dissection and the risk of recurrence of stroke. Based on imaging evidence, it is important to develop individualized treatment strategies for different risk groups. Currently, High-resolution magnetic resonance vessel wall imaging (MR-VWI) technology has made significant progress in the qualitative diagnosis of eCAD, vascular lesion progression, and the assessment of recurring ischemic stroke risk. To better understand the pathogenesis and progression of eCAD using MR-VWI, a comprehensive review is presented here.

## Introduction

eCAD refers to the separation of the arterial wall structure between the intima and the media or the adventitia due to the flow of cervical artery blood into the vessel wall through a damaged arterial intima, or bleeding in the nutrient vessels of the arterial wall, leading to the formation of intramural hematoma. This can cause stenosis, occlusion of the lumen, or the formation of dissecting aneurysms (4). eCAD includes both carotid artery dissection and vertebral artery dissection.

Although the majority of eCAD cases have a benign clinical course, it remains the second leading cause of stroke in young and middle-aged adults, second only to atherosclerosis (1). About half of eCAD-related ischemic strokes or transient ischemic attacks occur within 2 weeks of eCAD formation (5). Clinical management strategies for eCAD are continuously being explored and optimized, as revealed by the recently published CADISS and TREAT-CAD studies (2, 3). There is still controversy in the latest guidelines regarding the optimal duration of anti-thrombotic therapy after the acute phase, the use of statins, and the selection of the best vascular interventional treatment plan (6–9). Pathologically, eCAD-related vascular lesions have the characteristic of continuous change over time, including recanalization after occlusion, normalization of blood flow after previous stenosis or dissecting aneurysm, and chronic stenosis or occlusion. Early diagnosis and treatment can effectively prevent the occurrence of stroke, thus reducing or avoiding neurological dysfunction caused by stroke (10). Different diagnostic and therapeutic strategies at different time points may impact the progression of vascular lesions.

The traditional imaging methods used to evaluate eCAD are mainly conventional luminal imaging techniques, including carotid ultrasound, computed tomography angiography (CTA), magnetic resonance angiography (MRA), and digital subtraction angiography (DSA) (11). These methods indirectly infer potential pathological changes in the lesion wall through abnormal luminal morphological features, which makes early qualitative diagnosis difficult. Additionally, abnormal vessel wall lesions may be missed when the luminal display appears normal, and relying on changes in luminal lesions may not accurately reflect treatment efficacy promptly (12). These factors increase the difficulty of diagnosing and evaluating the progression of eCAD. In contrast, high-resolution magnetic resonance vessel wall imaging (MR-VWI) can directly display intramural hematoma, intimal flaps, double-lumen signs, pseudoaneurysms, and mural thrombi, which helps qualitatively stage and classify suspected eCAD in clinical practice. MR-VWI is a powerful tool for eCAD diagnosis and evaluation (13–15). Given the incomplete understanding of the pathogenesis of eCAD and the lack of optimized and personalized treatment options, further understanding of the natural course of eCAD using MR-VWI and monitoring the healing efficacy of antiplatelet therapy may have important value. Here, we review the progress in research on the characteristics and prognostic evaluation of eCAD lesions using MR-VWI.

## MR-VWI techniques

Multiple studies on MR-VWI in the evaluation of extracranial carotid artery vascular disease have demonstrated the reliability of its diagnostic efficacy and significant correlation with corresponding tissue pathology results (16, 17). The 3D T1-weighted black-blood sequence has multiple advantages in the diagnosis of eCAD. Firstly, the 3D T1-weighted black-blood sequence provides a large field of view and full coverage of the head and neck blood vessels in a single acquisition, which is important for identifying the location and number of eCAD lesions. Secondly, the imaging time of the 3D T1-weighted black-blood sequence is relatively short, ensuring that the total scan time is within an acceptable range, while being insensitive to motion artifacts, ensuring image quality. Thirdly, the 3D T1-weighted black-blood sequence can be used for maximum intensity projection (MIP) and surface reconstruction, as well as maximum or minimum density projection reconstruction. The MIP can display the overall length and morphology of intramural hematoma in a three-dimensional manner, which helps to locate dissections, especially in segments with tortuous arterial courses (14). Finally, T1-weighted blackblood sequence has the advantages of safety and non-invasiveness, making it an important tool for clinical follow-up and suitable for large populations.

Suppression of flowing blood signal is key to black blood imaging techniques. Different black-blood techniques have their own advantages and disadvantages in improving vessel lumen-wall contrast and suppressing blood flow (18). One option is to use inflow saturation to add a saturation band in the direction of blood flow. However, this method is prone to motion artifacts and residual high-signal debris in the lumen. Black-blood double inversion recovery (DIR) technique involves two RF inversion pulses, one non-selective 180° inversion pulse and one spatially selective 180° pulse. The signal of flowing blood can be well suppressed by setting an appropriate inversion time (TI). Based on the DIR technique, the quadruple inversion recovery (QIR) technique has also been developed and applied (19). The QIR technique can effectively suppress blood flow signals in contrast-enhanced T1-weighted images, while the blood suppression effect of DIR is poorer due to the shortened T1 time of blood in T1-weighted images. DIR/QIR imaging techniques have good spatial resolution, signal-to-noise contrast, and fewer flow artifacts, but their drawback is that they have a longer acquisition time and are mostly used in 2D sequences. In recent years, various 3D imaging techniques have been developed for large field-of-view imaging, which can image both intracranial and extracranial carotid arteries, and significantly reduce imaging time (20). Under 3D isotropic voxel acquisition, multi-plane reconstruction (MPR) can be used to reconstruct the vessel wall structure of the carotid artery from multiple directions, especially valuable for tortuous segments of the cervical artery.

Variable flip angle repulsive pulse fast spin-echo sequence is one of the most common 3D MR-VWI sequences, using non-selective pulses and variable angle repulsive pulses, resulting in significantly shorter echo spacing and improved scanning efficiency (21). It has different names in different MRI scanner vendors, including CUBE [GE], SPACE [Siemens], and VISTA [Philips] (20). The three-dimensional motion-sensitized driven equilibrium prepared rapid gradient echo (3D MERGE) sequence is based on the flow-independent motion-sensitized driven equilibrium (MSDE) pre-pulse technique. Recent studies of the 3D MERGE sequence have shown that MR-VWI can provide additional signals, such as the complete collapse of thrombotic and occlusive arteries, which helps to explore the causes of stenosis or occlusion and the mechanism of ischemic stroke (22). Meanwhile, some sequences are sensitive to intraplaque hemorrhage and intramural hematoma, such as the 3D T1-weighted magnetization-prepared rapid acquisition gradient echo (3D T1-MPRAGE) sequence and the 3D simultaneous non-contrast angiography and intraplaque hemorrhage (3D SNAP) sequence (23). Previous studies have verified the high sensitivity and specificity of these sequences in identifying intraplaque hemorrhage or arterial dissection wall hematoma (24–26). Recently, a quantitative vascular wall T1 mapping technique has been applied in the evaluation of arterial wall lesions. This sequence can provide more accurate and repeatable quantitative indicators to identify arterial wall hemorrhagic components and monitor the evolution of hemorrhagic components (27, 28).

## 3D fast spin echo sequence (T1-SPACE/CUBE/VISTA)

The 3D fast spin echo sequence is a T1-weighted imaging technique based on fast spin echo technology, which has an inherent black blood effect and superior signal-to-noise ratio performance (21). Several studies have reported on the diagnostic value of the 3D T1-VISTA sequence in evaluating eCAD. All studies have shown that the 3D T1-VISTA sequence is more valuable than traditional luminal imaging techniques in the diagnosis of eCAD. The inter-group and intra-group consistency of the 3D T1-VISTA sequence evaluation is very good (29). A study based on the 3D T1-VISTA sequence demonstrated the diagnostic efficacy of this sequence in eCAD by identifying high signal between the walls, intimal flaps, double lumen sign, and tumorous dilatation, with a sensitivity and specificity of 95.2 and 100%, respectively. Takemoto et al. first reported the diagnosis of eCAD using the T1-VISTA sequence on a 1.5 T magnetic resonance scanner (30). This study showed that compared to the 2D spin echo T1-weighted imaging combined with a time-of-flight (TOF) MRA scanning scheme, T1-VISTA can improve the evaluation of intramural hematoma in the vertebral artery. Another related study showed that all patients with vertebral artery dissection had abnormal enhancement of the vessel wall in the contrast-enhanced T1-VISTA images (31). Similar findings were also observed in T1 black blood sequence studies from two different manufacturers (T1-CUBE and T1-SPACE) (14, 32). The combination of 3D T1-SPACE and a coil that can cover the head and neck blood vessels further demonstrates the value of MR-VWI in the diagnosis of eCAD (33). In addition, the 3D T1-SPACE sequence is relatively less sensitive to metallic artifacts caused by post-traumatic metal fixation, allowing the evaluation of vertebral arteries to be unaffected by the presence of metallic artifacts. However, it is very difficult to differentiate intramural hematoma from adherent thrombus solely based on the 3D T1 black blood sequence in cases of complete arterial occlusion without other typical signs (33).

## DANTE

The Delay Alternating with Nutation for Tailored Excitation (DANTE) pre-pulse 3D T1-SPACE is an improved black blood imaging technique that continuously applies low-angle excitation pulses combined with stable diffusion gradients to produce varying signal intensities between dynamic blood and stationary tissue, thus suppressing blood flow signal (34). Compared to traditional 3D T1-SPACE black blood sequences, it significantly improves the suppression of arterial and venous lumen signals. DANTE-SPACE enables simultaneous imaging of the head and neck arteries with high time efficiency and the ability to assess the range and burden of arterial lesions (34). Lee K et al. used the DANTE-SPACE sequence to demonstrate short-term follow-up changes in a patient with eCAD (35). MIP and large scan field of view can simultaneously image the head and neck arteries. Submillimeter high-resolution images combined with MIP are very helpful for assessing intimal flaps in eCAD. Secondly, the DANTE-SPACE sequence has uniform and stable blood flow signal suppression ability and is not affected by blood flow velocity. Slow blood flow and turbulence at arterial stenoses often reduce the effectiveness of black blood flow suppression techniques. Previous MP-RAGE sequences using various blood flow signal suppression techniques such as DIR and MSDE techniques are affected by slow blood flow (34). The DANTE-SPACE sequence is not affected by this. Thirdly, the DANTE-SPACE sequence is a spin echo sequence with uniform fat suppression ability and a higher signal-to-noise ratio, which is insensitive to motion and magnetic susceptibility artifacts (34). Based on the above advantages, the DANTE-SPACE sequence can non-invasively and dynamically detect changes in eCAD.

## SNAP

The SNAP sequence is an imaging sequence that uses a selective phase-sensitive inversion recovery technique to simultaneously visualize bright and dark blood (23). The SNAP sequence has significant advantages in diagnosing etiologies such as intraplaque hemorrhage and intramural hematoma (23, 36, 37). Several studies have shown that non-contrast-enhanced MRA images based on SNAP sequence imaging have good consistency with 3D TOF-MRA and contrast-enhanced MRA in evaluating arterial luminal stenosis (23, 36, 38). With 3D multi-echo and gradient-echo (MERGE) images as the reference standard, studies have found that SNAP and traditional wall imaging sequences have good consistency in evaluating luminal stenosis and intramural hematoma in patients with eCAD (37). In addition, previous studies have used the 3D SNAP sequence to image the vessel wall and lumen of eCAD lesions, which takes about half the total time of 3D TOF-MRA combined with the T1W black blood sequence. Through phase-sensitive reconstruction, the 3D SNAP sequence can obtain optimized luminal signals and high signal contrast to display intramural hematoma. The 3D SNAP sequence may have advantages over the above black blood sequence as it can provide MRA and vascular wall imaging in a single scan (23). Another study compared SNAP with traditional MRI sequences and carotid ultrasound and found that SNAP had better efficacy in detecting intramural hematoma and had higher sensitivity and specificity in diagnosing eCAD (24). Moreover, by analyzing the absolute signal intensity and signal intensity index, SNAP and T1W black blood sequence can reflect the changes of intramural hematoma after treatment (24). There was no significant difference in the absolute signal intensity and signal intensity index of intramural hematoma between SNAP and T1W black blood sequence images, but there were differences in the absolute signal enhancement and signal intensity index before and after treatment, indicating that SNAP and T1W black blood sequence have similar value in evaluating intramural hematoma after treatment (24). The vessel wall images obtained by 3D SNAP sequence have the characteristics of heavy T1 weighting, fast acquisition, large longitudinal field of view, and isotropic high resolution. 3D SNAP is recommended as an ideal and fast MR imaging technique that can reliably identify eCAD, especially for tortuous cervical arteries and long-segment lesions (37, 39).

## MR-VWI features of eCAD

The MR-VWI characteristics of eCAD include typical signs such as intramural hematoma, pseudoaneurysm, elongated or tapered stenosis, intimal flap, and double lumen sign, as well as occlusion above the bifurcation of the carotid artery by more than 2.0 cm with concomitant pseudoaneurysm (40). Utilizing the MR-VWI technique to evaluate the location, degree of luminal stenosis, and abnormal changes of the vessel wall in eCAD may offer advantages based on the suppression of intraluminal blood flow signal.

### Intimal flap or double-lumen sign

The intimal flap or double lumen sign refers to the arterial blood flow being separated by a torn intimal septum into two lumens, true and false. The strip-like signal between the true and false lumens or between the true lumen and intramural hematoma is the direct sign of arterial dissection. Double lumen sign (OR = 5.75, *p* = 0.018) indicates active dissection and potential luminal compromise (41). The sensitivity and specificity of MR-VWI in detecting intimal flaps or identifying double-lumen signs are significantly higher than those of traditional luminal imaging techniques (33, 42). The Borgess classification proved that dissections without intimal tear (type I) had a higher rate of healing than dissections with intimal tear (type II) after antithrombotic therapy, which guides the medical management of spontaneous CAD (43). MR-VWI with gadolinium can show varying degrees of contrast enhancement. Strong, homogeneous enhancement is often seen in acute dissections, while heterogeneous enhancement may indicate chronic changes (44). Meanwhile, Quantitative analysis of contrast-enhanced MR-VWI predicted the instability in unruptured CADs (45). Therefore, quantification of changes in vessel wall enhancement by MR-VWI provided important information for CCAD staging and risk stratification.

### Intramural hematoma and its evolution

Intramural hematoma and its temporal evolution mechanism involve the tearing of the arterial intima, leading to the entry of intraluminal blood into the vessel wall, or rupture of vasa vasorum within the arterial wall, both of which can cause stenosis or occlusion of the lumen (4). Two pathological mechanisms have been identified in eCAD: the first is the intimal tear type, in which the intraluminal blood flows into the false lumen due to the rupture of the arterial intima; the second is the vasa vasorum rupture type, in which the intramural hematoma is formed by the rupture of vasa vasorum in the arterial wall (46–49). The overall intramural hematoma detection rate on 3D- T1WI was >50% and peaked in 1–2 weeks (50). Intramural hematoma was a frequently detectable finding for the diagnosis of IAD compared to other radiological findings. MR-VWI sequences can be used to estimate the onset of eCAD by comparing the signal intensity of intramural hematoma, which changes with its evolution (51). According to the formation period of intramural hematoma, it can be displayed as mild, moderate, and high signal intensity (51). Chu et al. found a good correlation between MR-VWI features and histopathological features and were able to stage cervical artery intramural hematoma based on T1W and T2W image features (52). Within 3 days of onset, intramural hematoma appears as an iso-signal intensity on T1WI. When intramural hematoma appears as a high signal intensity on T1WI, the onset time may be around 1 week. Within 3 days to 2 months after onset, the thrombus appears as a high signal intensity on T1WI. In contrast, intramural hematoma appears as a high signal intensity within 2 months. Within 6–12 months after onset, the signal intensity of intramural hematoma is similar to that of adjacent structures, appearing as an iso-signal intensity. In addition, the homogeneity of the intramural hematoma signal can indicate whether it is a single or recurrent hemorrhage (51). For example, the homogeneous signal of intramural hematoma suggests a single rupture, while a mixed signal suggests recurrent hemorrhage or different rates of hemoglobin degradation. The classification of intramural hematoma in eCAD based on MR-VWI can help to further understand the pathophysiological mechanism of arterial dissection (51). Finally, based on the signal characteristics of intramural hematoma on MR-VWI, eCAD patients can be stratified for risk assessment, which can effectively prevent recurrent stroke or transient ischemic attack, especially in patients with atypical clinical symptoms. The presence of an intramural hematoma with high T1-weighted intensity is indicative of acute dissection (51). Antiplatelet therapy and anticoagulation may be considered in the acute phase to prevent further thrombus formation and propagation. In cases where the hematoma is large or associated with significant luminal narrowing, endovascular stenting or surgical reconstruction may be required to restore blood flow (53, 54).

### Arterial perivascular edema

Non-specific inflammatory-mediated arterial wall injury is the mechanism of spontaneous eCAD (55). Previous studies have shown that abnormal signals exist in the surrounding tissue of the diseased artery in spontaneous eCAD patients on MR-VWI images, indicating arterial perivascular edema (56). This signal change may be due to focal arterial wall inflammation or wall hematoma compression. However, perivascular arterial edema is more common in spontaneous eCAD patients, while traumatic eCAD is less common, although the latter has relatively larger wall hematomas. This negates the idea that perivascular edema around dissection is due to simple mechanical compression (56). A study comparing the incidence of perivascular edema in spontaneous eCAD and traumatic eCAD patients found that perivascular edema around wall hematomas was more common in spontaneous eCAD (56). This phenomenon helps to distinguish the mechanism of eCAD occurrence.

### Intraluminal thrombus

eCAD patients have a high risk of ischemic stroke in the early stages of the disease, and thrombus-induced embolism is the most common mechanism of post-eCAD ischemic stroke (57). Endothelial flaps and irregular surfaces may reflect endothelial damage, thereby activating thrombus formation, ultimately leading to occlusion of the diseased artery or embolism in distant arteries (58). Routine clinical use of antithrombotic drugs for eCAD patients, early diagnosis of eCAD, and clarification of the presence of attached wall thrombus can help explore the mechanism of eCAD occurrence and predict the risk of ischemic stroke. Timely and appropriate clinical treatment is crucial.

### eCAD-related ischemic stroke

eCAD-related ischemic stroke has a significant correlation with thrombus formation signs identified by imaging (42, 59). The use of a 3.0 T MRI scanner for 3D T1-SAPCE sequence scanning can have certain diagnostic values for attached wall thrombus in eCAD (33). The sensitivity and accuracy of MR-VWI in identifying attached wall thrombus in eCAD were 97.4 and 79.0%, respectively, with DSA as the reference standard. At the same time, the attached wall thrombus appearing on MR-VWI is closely related to local ischemic stroke (OR: 30.0; 95% confidence interval: 9.1–98.4; *p* < 0.001). Coppenrath et al. found that 2D T1-weighted enhanced sequences could display intraluminal attached wall thrombus formation in a study of 33 spontaneous eCAD patients, which was significantly correlated with ischemic stroke in the corresponding blood supply area (OR, 32.0; 95% CI: 3.6–281, 60). T1-SPACE provides a panoramic view of the dissection artery and can obtain the local and distant arterial thrombus load of the affected artery in one imaging acquisition. 3D-SPACE can more directly identify the presence of intraluminal blood clots in diseased arteries than DSA and is easier to identify multiple clots (33). High-resolution magnetic resonance imaging might give insights into pathogenesis of ischemic stroke in eCAD (42). The presence of irregular surface and intraluminal thrombus were related to stroke occurrence in patients with eCAD. Presence of intraluminal thrombus (OR = 5.56, *p* = 0.004) strongly correlates with ischemic stroke risk due to thromboembolism. Anticoagulation (e.g., heparin/warfarin or direct oral anticoagulants) is prioritized to prevent thrombus propagation and embolization, especially in acute phases (53). In certain circumstances, such as the presence of unstable thrombus in the vessel wall or further worsening of the degree of stenosis, EVT still need to be considered (60). Although the formation of dissecting aneurysms increases the risk of thromboembolism or further enlargement with obliteration of the vessel, leading to ischemic stroke or aneurysm rupture. Data from the prospective CADISS study suggest that carotid dissecting aneurysm is a relatively common sequela of extracranial vascular entrapment with a favorable prognosis and that the presence of dissecting aneurysm does not indicate a higher risk of recurrent stroke in patients with dissection (61). There was no difference in the persistence of dissecting aneurysm or development of new DA for antiplatelet vs. anticoagulant therapy (60, 61).

### Predictive indicators for risk of ischemic stroke in eCAD

Cerebral infarction caused by ischemia in eCAD is mainly due to embolism, which forms small clots at the site of intimal tears in arterial dissection (62). A study has shown that wall hematoma, irregular intimal surface, mural thrombus, and severe stenosis are significantly correlated with the occurrence of ischemic stroke in patients undergoing carotid artery examination (42). Multivariate regression analysis suggests that irregular intimal surface and mural thrombus are independent risk factors for acute ischemic stroke. Previous studies have shown that enhancement of the lesion lumen on MR-VWI indicates the formation of mural thrombus, which has a significant correlation with ischemic symptoms (63). However, another study found that high-signal foci within the dissection artery lumen were more common in patients with eCAD and ischemic stroke (44.3% versus 4.5%, *p* < 0.001), suggesting the formation of fresh mural thrombus (42). The MR-VWI findings in this study support the results of previous studies, indicating that embolism is the main mechanism of ischemic stroke in eCAD (58). Irregular intimal surface is another high-risk feature for the occurrence of ischemic stroke (42). Similarly, studies of carotid artery atherosclerotic plaques have shown that irregular intimal surface is one of the risk factors for thrombosis formation and is significantly associated with an increased risk of transient ischemic attack/ischemic stroke (64, 65). Irregular intimal surface in eCAD indicates intimal damage, which can lead to the formation of microthrombi and ultimately result in ischemic stroke.

## Issues and prospects

MR-VWI can cover a wide range of head and neck arteries in a single acquisition, showing the characteristic features of arterial wall and lumen, and has high black blood contrast. Unlike traditional luminal imaging techniques, MR-VWI can directly display typical signs of eCAD, such as wall hematoma, double lumen sign, and intimal flap, which can improve diagnostic confidence. Secondly, the identification of high-risk imaging features such as mural thrombus can help explore the mechanism of eCAD and predict the risk of ischemic stroke, which is important for a better understanding of the relationship between unstable eCAD, thrombosis formation and embolism occurrence, and timely and appropriate clinical treatment to optimize the diagnosis and treatment strategies for eCAD patients. Although MR-VWI has many advantages and potential for development, it is still a time-consuming technique. In addition, carotid artery imaging often requires a special surface coil, which may limit the widespread application of this imaging modality. It is believed that with the development of black blood techniques, MR-VWI will become a very promising imaging modality for the precise evaluation of eCAD.
